# Supplementary material for: Effectiveness and Safety of Ayurvedic Medicines in Type 2 Diabetes Mellitus Management: A Systematic Review and Meta-Analysis
Source: Front Pharmacol. 2022 Jun 8;13:821810. doi: 10.3389/fphar.2022.821810 (PMC9213670; doi:10.3389/fphar.2022.821810)
Supplement: Supplementary file 1 [file DataSheet1.zip › Supplementary Material/Forest and Funnel Plots/Zingiber officinale/HDL-C.pdf]

Zingiber officinale - HDL-C

Study	Intervention N	Intervention Mean	Intervention SD	Comparator N	Comparator Mean	Comparator SD	Mean Diff. with 95% CI	Weight (%)
Mahluji 2013	28	40.60	6.40	30	43.90	7.10	-3.30 [-6.79, 0.19]	15.88
Arablou 2014a, Arablou 2014b	33	46.20	8.30	30	44.20	7.60	2.00 [-1.94, 5.94]	14.93
Azimi 2014, Azimi 2016	41	58.09	4.93	39	51.53	9.49	6.56 [3.27, 9.85]	16.29
Mozaffari-Khosravi 2014, Talaei 2017, Talaei 2018	40	44.07	10.29	41	41.95	7.44	2.12 [-1.78, 6.02]	15.02
Arzati 2017, Zarezadeh 2018	23	42.80	7.80	22	44.30	8.60	-1.50 [-6.29, 3.29]	13.21
Carvalho 2020	47	50.30	10.90	56	48.88	10.10	1.42 [-2.64, 5.48]	14.69
Gholinezhad 2020	21	54.09	9.68	21	45.71	12.04	8.38 [1.77, 14.99]	9.99
**Overall**							2.01 [-0.90, 4.92]	

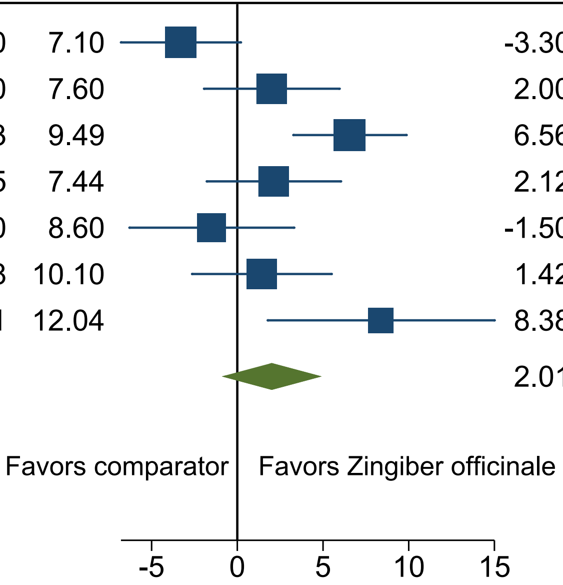

Heterogeneity: $\tau^2 = 10.75$, $I^2 = 71.38\%$, $H^2 = 3.49$

Test of $\theta_i = \theta_j$: $Q(6) = 21.94$, $p = 0.00$

Test of $\theta = 0$: $z = 1.35$, $p = 0.18$

Random-effects REML model
